# Linalool odor‐induced analgesia is triggered by TRPA1-independent pathway in mice

**DOI:** 10.1186/s12993-021-00176-y

**Published:** 2021-04-26

**Authors:** Hideki Kashiwadani, Yurina Higa, Mitsutaka Sugimura, Tomoyuki Kuwaki

**Affiliations:** 1 Department of Physiology, Graduate School of Medical and Dental Sciences, Kagoshima University, 8-35-1 Sakuragaoka, Kagoshima, 890-8544 Japan; 2 Department of Dental Anesthesiology, Graduate School of Medical and Dental Sciences, Kagoshima University, 8-35-1 Sakuragaoka, Kagoshima, 890-8544 Japan

**Keywords:** Linalool, Analgesia, TRPA1, TRPA1-deficient mice, TRPA1-selective antagonist

## Abstract

We had recently reported that linalool odor exposure induced significant analgesic effects in mice and that the effects were disappeared in olfactory-deprived mice in which the olfactory epithelium was damaged, thus indicating that the effects were triggered by chemical senses evoked by linalool odor exposure. However, the peripheral neuronal mechanisms, including linalool receptors that contribute toward triggering the linalool odor-induced analgesia, still remain unexplored. In vitro studies have shown that the transient receptor potential ankyrin 1 (TRPA1) responded to linalool, thus raising the possibility that TRPA1 expressed on the trigeminal nerve terminal detects linalool odor inhaled into the nostril and triggers the analgesic effects. To address this hypothesis, we measured the behavioral pain threshold for noxious mechanical stimulation in TRPA1-deficient mice. In contrast to our expectation, we found a significant increase in the threshold after linalool odor exposure in TRPA1-deficient mice, indicating the analgesic effects of linalool odor even in TRPA1-deficient mice. Furthermore, intranasal application of TRPA1 selective antagonist did not alter the analgesic effect of linalool odor. These results showed that the linalool odor-induced analgesia was triggered by a TRPA1-independent pathway in mice.

## Introduction

We had recently demonstrated that odor exposure of linalool (3,7-dimethylocta-1,6-dien-3-ol), one of the monoterpene alcohols found in lavender extracts, induces analgesic effects in mice [1]. We observed that the effects were disappeared in olfactory-deprived mice in which the olfactory epithelium was damaged, thus indicating that the effects were triggered by chemical senses evoked by linalool odor exposure. However, the peripheral neuronal mechanisms, including linalool receptors that contribute toward triggering the linalool odor-induced analgesia, have not yet been explored.

Odorous volatile compounds inhaled into the nostril are detected by two sensory systems, the main olfactory system and the trigeminal sensory system. In the main olfactory system, olfactory sensory neurons expressing a given odorant receptor is translated into electrical signals. These signals are then transmitted to the main olfactory bulb, the first relay station of the main olfactory system, and further to the olfactory cortices to perceive the olfactory input [2, 3]. In addition to the classical main olfactory pathway, the trigeminal nerve pathway contributes to the detection of odorous compounds in the nostril [4]. The ethmoidal nerve arising from the ophthalmic division of the trigeminal nerve projects into the nasal cavity and makes free end terminals in the epithelium　[5–8]. The central projections of the ethmoidal nerve terminate on the superficial laminae of the medullary dorsal horn [9, 10] with collateral branches which reach directly to the olfactory bulb [7, 11]. In spite of the individual peripheral receptors, the olfactory and trigeminal input mutually affect each other in the perception of odors [12–15], indicating that the information detected by the two chemosensory systems is functionally integrated in our central nervous system. The exact location of the interaction between the two systems is not yet determined. But several anatomical areas such as the olfactory epithelium, the olfactory bulb, the mediodorsal thalamus, the piriform cortex, the orbitofrontal cortex and the insula cortex have been proposed as the site [5, 7, 11, 16–18].

Chemosensors expressed on the free end terminals can be activated by the chemical compounds that are inhaled into the nostril and drive the trigeminal pathway [19]. Among the chemosensors, the transient receptor potential ankyrin 1 (TRPA1), which was first identified as the thermosensor [20], detects a range of odorous compounds [21], and plays a key role in triggering the allyl isothiocyanate odor-induced bradypnea in mice [22]. In addition to pungent odorous compounds, linalool activates the TRPA1 in dissociated dorsal root ganglia neurons and in human embryonic kidney 293 cells expressing the wild-type TRPA1 channels [23, 24]. These observations raise a hypothesis that the TRPA1 on the trigeminal nerve terminal in the nasal cavity detects the inhaled linalool and triggers the analgesic effects induced by linalool odor. To investigate this hypothesis, we measured the threshold for the behavioral pain response immediately after linalool odor exposure and compared the threshold between TRPA1-deficient (KO) mice and wild-type mice.

## Materials and methods

### Animals and housing conditions

Male wild-type (WT) mice (C57BL/6, weighing 26.6–34.6 g, *n* = 77) and TRPA1 KO mice (weighing 22.7–27.4 g, *n* = 43) were used in this study. The mutant mice were originally purchased from the Jackson Laboratory and genotyped as previously described [25]. They were maintained as heterozygotes in our facility and crossed to obtain null mutants and WT littermates. The mutant mice were backcrossed with C57BL/6J mice (Clea Japan Inc., Tokyo, Japan) for more than 10 generations. All animals were maintained under a constant temperature (24 °C ± 1 °C) with free access to food and water. The animals were housed with lights on at 7:00 A.M. and off at 7:00 P.M. All experiments were conducted during the light cycle, between 1:00 P.M. and 5:00 P.M. The animals were naive to linalool odor, and each mouse was used only once to avoid carryover effects.

### Chemicals

Linalool (CAS#: 78-70-6) was purchased from Tokyo Chemical Industry (Tokyo, Japan), stored at 4 °C, and dispensed into a glass vial during each trial to prevent degradation. A TRPA1 selective antagonist AP18 ((Z)-4-(4-chlorophenyl)-3-methylbut-3-en-2-oxime, gifted from Prof. Mori at Kyoto University [26]) was dissolved in polyethylene glycol (PEG#400, Nacalai, Kyoto, Japan) at 60 mM.

### Linalool odor exposure

We used a custom-made olfactometer for linalool odor exposure as described previously [1]. Briefly, 0.5 mL of linalool (> 96 %) was dispensed into an uncapped glass vial (diameter: 27.5 mm, content: 20 mL). The vial was placed in an odor chamber (0.32 L), and then linalool was vaporized at room temperature (24 °C ± 1 °C). Clean air deodorized using a charcoal filter and double-distilled water was introduced into the odor chamber (top diameter: 8 cm, base diameter: 11.5 cm, height: 15 cm, content: 1 L) at a constant flow rate (1 L/min). After 20 min of pre-ventilation of the linalool odor, a mouse was placed in the observation chamber and exposed to linalool odor for 5 min. Because the humidity of the carrier gas and the temperature of the odor chamber were maintained constant, the concentration of linalool odor was considered to be constant.

### Acclimatization for pain assay

For acclimatization to the experimental condition, the animals were moved to the experiment room for 2 h, handled and gently touched for 3 min, and their head and body were covered with a towel and loosely restrained for 3 min. This series of acclimatization procedures was repeated for 6 days. On the experiment day, the mice were moved to the experiment room 2 h before the experiment.

### Tail pincher test

For evaluating the analgesic effects of linalool odor, we measured the mechanical nociceptive threshold for tail pinch using calibrated forceps (Rodent Pincher-analgesia meter, Bioseb, Pinellas Park, USA) [27, 28]. Immediately after 5 min of odor exposure, we gently restrained the mouse with a towel and pressured on the marking of the tail using the calibrated forceps. We recorded the latency of the flicking, tail withdrawal, or struggling of the mouse in the cotton towel. We repeatedly measured the threshold for five times (trial interval: 10–15 s). We then excluded the maximum and minimum values from the sets of five measurements and used the average value of another three trials as the threshold value [28]. To prevent the mouse from being injured, a cut-off pressure point was set at 500 g. Each animal was used only once to prevent hyperalgesia.

### Hot plate test

To assess the thermal nociceptive threshold, we performed a classical hot plate test with electronically controlled hot plate apparatus (#7280; Ugo Basile, Italy) as previously reported [1]. We set the hot plate temperature at 54.5 °C. Immediately after 5 min odor exposure, we placed a mouse on the hot plate. The latency before the animal licked, shock, or fluttered its hind paw, or jumped on the hot plate was recorded. To prevent the mouse from being injured, a cutoff time was set to 1 min.

### Intranasal application of TRPA1 antagonist

To prevent the function of TRPA1 in the nostril, we administered the TRPA1 antagonist into the nasal cavity as previously described [21]. A small ball of the TRPA1 solution (10 µL) was attached to the nostril and was aspirated into the nasal cavity with spontaneous breathing. For the negative control experiment, a same amount of vehicle solution was administered. The prevention of TRPA1 was confirmed by the behavioral odor preference/avoidance test. 10 min after the intranasal application, further behavioral pain tests were performed.

### Data analyses

GraphPad Prism 8 (GraphPad Software, San Diego, CA, USA) was used for statistical analyses. Bartlett’s test was applied to examine the equal variances among groups. The difference between each pair of groups was compared using Welch’s ANOVA, followed by Dunnett’s T3 multiple comparison test. To avoid false-negative results caused by the effects of meaningless pairs ((WT) / control (CON) vs. TRPA1-deficient mice (KO) / linalool (LIN) and WT / LIN vs. KO / CON)), we selected four pairs (WT / CON vs. WT / LIN, WT / CON vs. KO / CON, KO / CON vs. KO / LIN, WT / LIN vs. KO / LIN) among the possible six pairs. Differences with *p* values < 0.05 were considered to be significant. We also calculated Cohen’s d for comparison of two groups as the effect size. The effect size was considered as large in case of d > 0.8, medium in case of d > 0.5, and small in case of d > 0.2. The datasets used and/or analyzed during this study are available from the corresponding author on reasonable request.

## Results

### Linalool odor-induced analgesia in TRPA1 KO mouse

**Fig. 1:**
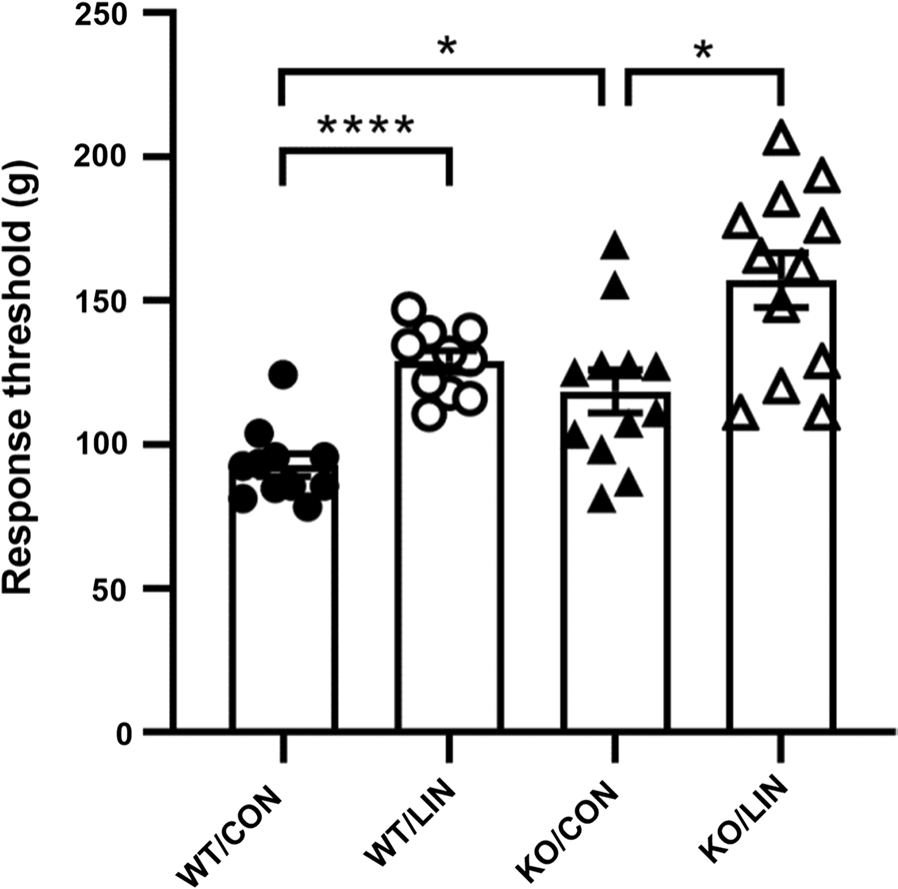
Linalool odor-induced analgesia for mechanical nociception in TRPA1-deficient mice. Behavioral response threshold for mechanical nociceptive stimulation to tail was plotted. WT: wild-type mice; KO: TRPA1-deficient mice; CON: odorless air-exposed control mice; LIN: linalool odor-exposed experimental mice. Bars represent mean ± SEM. *n*_WT/CON_ = 11, *n*_WT/LIN_ = 10, *n*_KO/CON_ = 12, *n*_KO/LIN_ = 12, ****p < 0.0001, *p < 0.05 (post hoc Dunnett’s T3 multiple comparison test)

To investigate the contribution of TRPA1 to the linalool odor-induced analgesia, we measured the behavioral response threshold for mechanical nociceptive stimulation using the tail pincher test [27, 28] immediately after linalool odor exposure (Fig. 1). Bartlett’s test revealed the significant difference in variances among the four group (WT / CON, WT / LIN, KO / CON, KO / LIN) (*χ*^2^ = 13.63, *p* = 0.0035). Welch’s ANOVA, followed by Dunnett’s T3 multiple comparison test, was applied to compare the difference in each pairs as an alternative method of ordinary two-way ANOVA followed by Sidak’s test. The results of Welch’s ANOVA showed that a significant difference existed among the groups (*W*_3, 22.29_ = 20.87, *p* < 0.0001). The post hoc Dunnett’s T3 multiple comparison test indicated that linalool exposure induced a significant increase in the response threshold (*θ*) in wild-type mice (*θ*_WT/CON_ = 92.92 ± 3.846 g, *θ*_WT/LIN_ = 128.8 ± 3.741 g, *p* < 0.0001, *d* = 2.909) and also in TRPA1-deficient (KO) mice with large effect sizes (*θ*_KO/CON_ = 118.5 ± 7.482 g, *θ*_KO/LIN_ = 157.1 ± 9.503 g, *p* = 0.0171, *d* = 1.302). The threshold of KO mice after odorless air exposure was significantly larger than that of WT mice with a large effect size (*p* = 0.0297, *d* = 1.235), indicating that the basal response threshold for mechanical nociception was higher in KO mice. Moreover, the threshold after linalool odor exposure was slightly (but not significantly) increased in KO mice (*p* = 0.0557, *d* = 1.103).

**Fig. 2:**
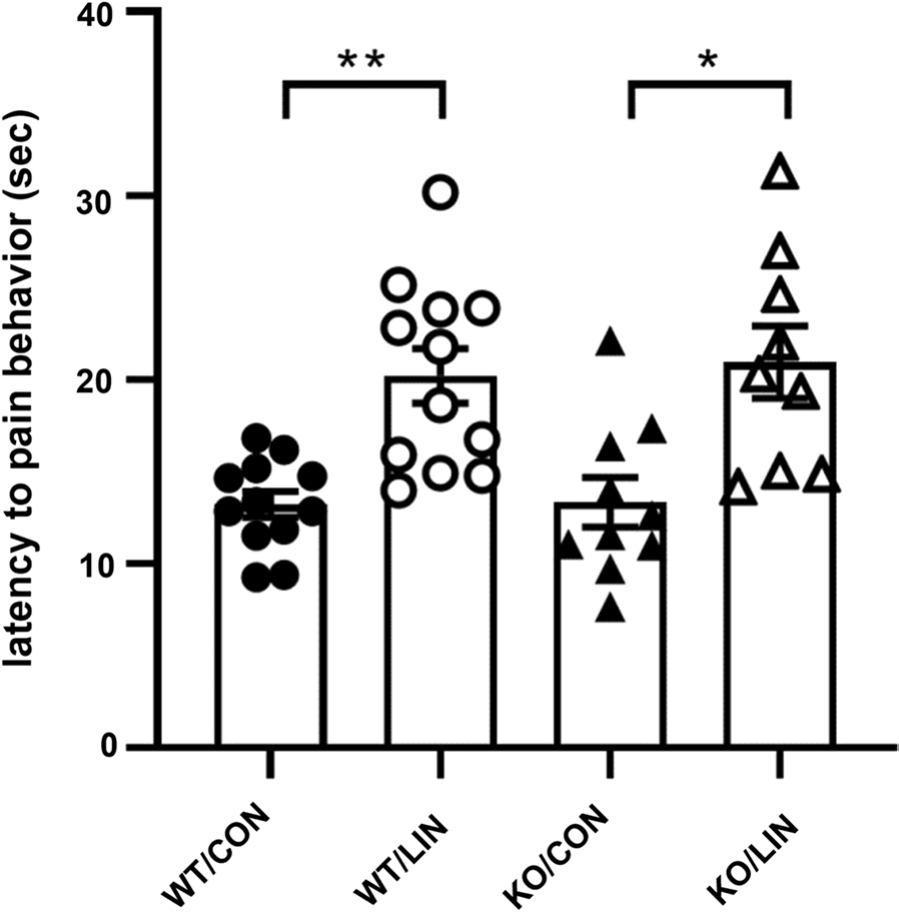
Linalool odor-induced analgesia for thermal nociception in TRPA1-deficient mice. Behavioral response latency for thermal nociceptive stimulation to hind paw was plotted. WT: wild-type mice; KO: TRPA1-deficient mice; CON: odorless air-exposed control mice; LIN: linalool odor-exposed experimental mice. Bars represent mean ± SEM. *n*_WT/CON_ = 12, *n*_WT/LIN_ = 12, *n*_KO/CON_ = 10, *n*_KO/LIN_ = 9, **p < 0.005, *p < 0.05 (post hoc Dunnett’s T3 multiple comparison test)

Next, we examined the analgesic effects in thermal nociceptive stimulation with the hot plate test in TRPA1 KO mice (Fig. 2). The hot plate test revealed that the thermal nociceptive threshold was significantly increased in both wild type mice and the KO mice after linalool odor exposure as in the tail pincher test. Bartlett’s test revealed that there were relatively large (but not significant) differences in variances among the four group (WT / CON, WT / LIN, KO / CON, KO / LIN) (*χ*^2^ = 7.305, *p* = 0.0614). The results of Welch’s ANOVA showed that a significant difference existed among the groups (*W*_3, 18.94_ = 9.118, *p* < 0.0006). The post hoc Dunnett’s T3 multiple comparison test indicated that linalool exposure induced a significant increase in the response latency (*λ*) in wild type mice (*λ*_WT/CON_ = 13.22 ± 0.704 s, *λ*_WT/LIN_ = 20.21 ± 1.481 s, *p* < 0.0023, *d* = 1.741) and also in TRPA1 KO mice (*λ*_KO/CON_ = 13.34 ± 1.347 s, *λ*_KO/LIN_ = 20.95 ± 1.973 s, *p* = 0.0251, *d* = 1.490) with large effect sizes. The response latency of KO mice after odorless air exposure was not significantly larger than that of WT mice with a small effect size (*p* > 0.9999, *d* = 0.036), indicating that the basal response latency for thermal nociception was not different between phenotypes. In addition, the latency after linalool odor exposure was not significantly increased in KO mice (*p* = 0.9966, *d* = 0.135). These results indicate that linalool odor exposure induced significant analgesic effects even in TRPA1 KO mice.

### Linalool odor-induced analgesia under pharmacological prevention of intranasal TRPA1

**Fig. 3:**
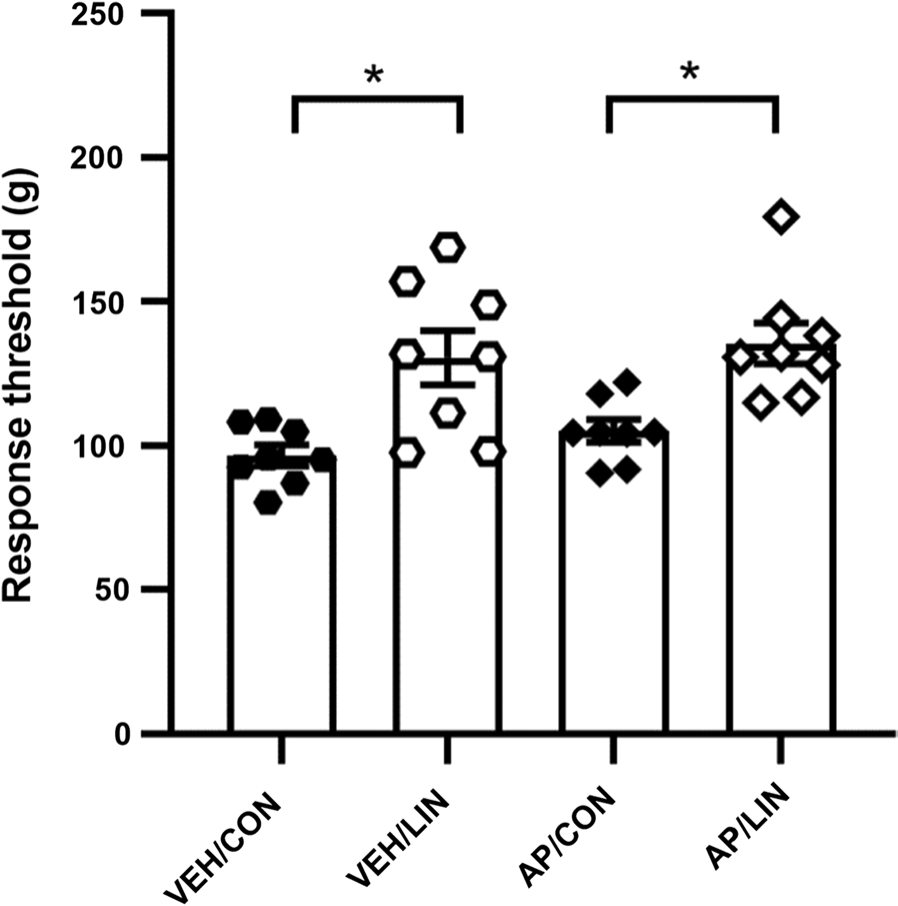
Linalool odor-induced analgesia after intranasal application of TRPA1-selective antagonist. Behavioral response threshold for mechanical nociceptive stimulation to tail was plotted. VEH: intranasal vehicle-treated mice; AP: intranasal AP18 (TRPA1 selective antagonist)-treated mice; CON: odorless air-exposed control mice; LIN: linalool odor-exposed experimental mice. Bars represent mean ± SEM. *n*_VEH/CON_ = 8, *n*_VEH/LIN_ = 8, *n*_AP/CON_ = 8, *n*_AP/LIN_ = 8, *p < 0.05 (post hoc Dunnett’s T3 multiple comparison test)

To examine the possibility that the other receptor(s) may compensate the linalool response in TRPA1 KO mice, we accessed the linalool odor analgesia by pharmacological prevention of intranasal TRPA1 in wild type mice (Fig. 3). Intranasal application of AP18, a TRPA1 selective antagonist, revealed that the mechanical nociceptive threshold was not significantly altered after the TRPA1 antagonist administration. Bartlett’s test revealed the significant difference in variances among the four group (VEH / CON, VEH / LIN, AP / CON, AP / LIN) (*χ*^2^ = 8.250, *p* = 0.0411). The results of Welch’s ANOVA showed that a significant difference existed among the groups (*W*_3, 14.85_ = 9.282, *p* < 0.0011). The post hoc Dunnett’s T3 multiple comparison test indicated that linalool exposure induced a significant increase in the response threshold (*θ*) in vehicle-treated mice (*θ*_VEH/CON_ = 96.62 ± 3.639 g, *θ*_VEH/LIN_ = 130.5 ± 9.464 g, *p* < 0.0314, *d* = 1.672) and also in AP18-treated mice (*θ*_AP/CON_ = 105.1 ± 3.887 g, *θ*_AP/LIN_ = 135.5 ± 7.170 g, *p* = 0.0125, *d* = 1.867) with large effect sizes. The threshold of AP18-treated mice after odorless air exposure was not significantly different from that of WT mice (*p* = 0.4157, *d* = 0.794), indicating that the basal response threshold was not affected by the intranasal AP18 treatment. In addition, the threshold after linalool odor exposure was not significantly altered in AP18-treated mice (*p* = 0.987, *d* = 0.210).

Altogether, we concluded that TRPA1 did not contribute toward triggering the linalool odor-induced analgesia, and thus the analgesia was triggered by a TRPA1-independent pathway in mice.

## Discussion

We first hypothesized that the TRPA1 expressed on the trigeminal nerve in the nasal epithelium detects the linalool inhaled into the nostril and triggers the linalool odor-induced analgesia. To explore this hypothesis, we conducted a mechanical (tail pincher test) and a thermal (hot plate test) pain test immediately after linalool odor exposure in TRPA1KO mice. Our results, in contrast to our prediction, demonstrated that the KO mice exhibited a significant analgesic effect on both mechanical and thermal nociception. Furthermore, intranasal application of TRPA1 selective antagonist did not impair the linalool odor-induced analgesia. These results imply that the analgesic effect is triggered by a TRPA1-independent pathway.

We first intended to conduct the ordinary two-way ANOVA (genotype × odor treatment), followed by Sidak’s multiple comparison test. However, the results of Bartlett’s test revealed unequal variances among the examined groups, indicating that the ordinary two-way ANOVA was not applicable to our dataset. Therefore, we applied Welch’s ANOVA, followed by Dunnett’s T3 multiple comparison test [29]. Using these statistical methods, we could not examine the major effects (genotype, odor treatment, and its interaction), but we could compare the difference in each group pair.

In addition to TRPA1, other three types of TRP family channels (transient receptor potential vanilloid 1 (TRPV1), transient receptor potential vanilloid 2 (TRPV2) and transient receptor potential melastatin 8 (TRPM8)) are expressed on trigeminal ganglion cells (TRPV1 [30–32], TRPV2 [33, 34], TRPM8 [30, 35, 36]). In vitro studies have suggested that linalool could be also detected by TRPM8 [37], though the half maximal effective concentration (EC50) of TRPM8 is sixty times higher than that of TRPA1 [23, 37]. Therefore, the trigeminal system could contribute toward triggering the linalool odor-induced analgesia through TRPM8. Further studies are required to evaluate the contribution of TRPM8.

In our experimental condition, the KO mice exhibited a higher response threshold to mechanical pain stimulation. TRPA1 is expressed in not only trigeminal ganglion cells but also dorsal root ganglion cells with small diameter [20, 30] and could affect the noxious mechanical transduction [38]. Our result is consistent with previous studies indicating that TRPA1-deficient mice had a higher threshold to mechanical pain stimuli than that of wild type mice [25, 39].

In this study, we examined the contribution of TRPA1 to analgesic effects of linalool odor that is vaporized in room temperature with simple odor chamber system. However, the contribution of TRPA1 at higher linalool concentration has not yet untried. Previous studies have shown that the trigeminal system could be generally activated in higher odor concentrations in human [40, 41] and mouse [42]. Therefore higher concentration linalool might affect to the analgesic effects via trigeminal TRPA1 system. In addition to the concentration, the duration of linalool odor exposure could affect the results. Yousem and colleagues reported that repetitive odor stimulation of trigeminal nerve enhances the cortical responses in contrast to the desensitization of olfactory nerve input on human subjects [43]. Therefore the longer exposure could make increase the number of inhalation of linalool odor, which in turn might drive the trigeminal activation via TRPA1 and affect to the analgesic effects. Further studies are required to assess these points.

Except for the trigeminal system, linalool can also be detected by classical odorant receptors expressed on olfactory sensory neurons. To our knowledge, at least one odorant receptor (hOR1C1) has been reported to detect linalool [44–46]. However, hOR1C1 is present only in the genome of humans and not mice [47]. Therefore, it is possible that other unknown odorant receptors could detect linalool odor and trigger analgesic effects in mice.

## Data Availability

The dataset supporting the conclusions of this is available from the corresponding author on reasonable request.
